# The feasibility of modified HIV and antiretroviral drug testing using self-collected dried blood spots from men who have sex with men

**DOI:** 10.1186/s12879-021-06110-x

**Published:** 2021-05-05

**Authors:** Wei Luo, Vickie Sullivan, Pollyanna R. Chavez, Sarah E. Wiatrek, Maria Zlotorzynska, Amy Martin, Rebecca Rossetti, Travis Sanchez, Patrick Sullivan, Robin J. MacGowan, S. Michele Owen, Silvina Masciotra

**Affiliations:** 1 Centers for Disease Control and Prevention, National Center for HIV, Viral Hepatitis, STD, and TB Prevention, Division of HIV/AIDS Prevention, Laboratory Branch, 1600 Clifton Road, Mailstop US H-17, Atlanta, Georgia 30329 USA; 2 Emory University, Atlanta, GA USA

**Keywords:** Self-collected DBS, HIV testing, Viral load monitoring, MSM, Online recruitment

## Abstract

**Background:**

In the US, one in six men who have sex with men (MSM) with HIV are unaware of their HIV infection. In certain circumstances, access to HIV testing and viral load (VL) monitoring is challenging. The objective of this study was to evaluate the feasibility of conducting laboratory-based HIV and antiretroviral (ARV) drug testing, and VL monitoring as part of two studies on self-collected dried blood spots (DBS).

**Methods:**

Participants were instructed to collect DBS by self-fingerstick in studies that enrolled MSM online. DBS from the first study (*N* = 1444) were tested with HIV serological assays approved by the Food and Drug Administration (FDA). A subset was further tested with laboratory-modified serological and VL assays, and ARV levels were measured by mass spectrometry. DBS from the second study (*N* = 74) were only tested to assess VL monitoring.

**Results:**

In the first study, the mail back rate of self-collected DBS cards was 62.9%. Ninety percent of DBS cards were received at the laboratory within 2 weeks from the day of collection, and 98% of the cards had sufficient spots for one assay. Concordance between FDA-approved and laboratory-modified protocols was high. The samples with undetectable ARV had higher VL than samples with at least one ARV drug. In the second study, 70.3% participants returned self-collected DBS cards, and all had sufficient spots for VL assay. High VL was observed in samples from participants who reported low ARV adherence.

**Conclusions:**

In these studies, MSM were able to collect and provide adequate DBS for HIV testing. The FDA-approved and laboratory-modified testing algorithms performed similarly. DBS collected at home may be feasible for HIV testing, ARV measurement, and monitoring viral suppression.

## Background

An estimated 70% of new HIV diagnoses in the United States (US) and dependent areas in 2017 were from gay, bisexual and other men who have sex with men (MSM), while one in six MSM with HIV were unaware of their infection [1]. Despite annual HIV testing recommendations from the Centers for Disease Control and Prevention (CDC) [2], 30–40% of internet-recruited MSM reported not testing in the past year [3, 4].

HIV testing increases awareness of HIV status. Travel, clinic wait times, work schedules, and confidentiality concerns are commonly reported barriers to HIV testing [5]. HIV self-tests have provided an option for testing in a private and convenient setting; however, HIV self-testing only provides a preliminary result. Additional testing, such as HIV-1 viral load (VL), is required for health monitoring purposes when an HIV infection is diagnosed. Self-collected dried blood spot (DBS) cards returned by mail for laboratory testing might be an alternative option for HIV testing and VL monitoring when access to a clinical setting is challenging.

The objective of this study was to evaluate the feasibility of using DBS cards collected by MSM recruited online for HIV serology, antiretroviral (ARV) drug testing, and monitoring viral suppression.

## Methods

### Data and DBS collection

**Fig. 1:**
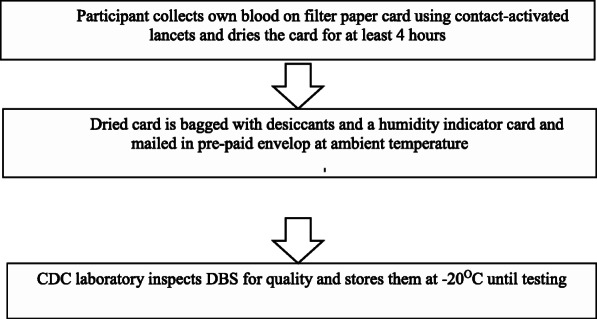
Dried Blood Spot Collection Flow Chart

The “Evaluation of HIV Self-Testing Among MSM Project” (eSTAMP) was a CDC funded study that evaluated the effect of providing self-testing kits on sexual risk behaviors, partner testing, and linkage to care after self-tested positive. The study recruited MSM online to participate in a formative study [6] in May–December 2014 and a randomized controlled trial (RCT) [4] in March 2015–December 2016. Eligibility for eSTAMP included male at birth and identified as male, ≥ 18 years old, residing in the US, HIV-negative or unaware of HIV status, able to read in English, anal sex with ≥1 man during the past 12 months, not on antiretroviral medications to prevent HIV, never in an HIV vaccine trial, and not having a bleeding disorder. In the formative study, after completing a baseline survey participants were mailed a study package containing one OraQuick In-Home HIV test (OraSure Technologies, Bethlehem, PA), a self-test version of Sure Check HIV 1/2 Assay (Chembio Diagnostic System Inc., Medford, NY) used under an Investigational Device Exemption from the Food and Drug Administration (FDA), and a DBS collection kit. Participants were asked to submit test results online after self-testing. In the one-year RCT, participants were required to complete not only baseline but also quarterly follow-up online surveys. They were mailed a study package after completing the12-month follow-up survey or reporting an HIV positive test result. All eSTAMP participants who received the study package were instructed to collect their own DBS sample within 48 h of self-testing. The DBS collection kit included written instructions, a web-link to a demonstration video, two Whatman 903 protein saver DBS cards (GE healthcare Bio-sciences, Pittsburgh, PA), two Microtainer contact-activated lancets (Becton Dickinson and Co., Franklin Lakes, NJ), two sterile gauze, two alcohol swabs, two bandages, three desiccants, a humidity indicator card (Poly Lam Products, Corp., Williamsville, NY), two gas-permeable plastic zipper seal bags, and a pre-paid return envelope. All participants provided electronic informed consent. Figure 1 illustrates the flow of DBS from collection to processing at the CDC laboratory.

The American Men’s Internet Survey (AMIS) study is an annual online behavioral surveillance survey of MSM residing in the US [7, 8]. Eligibility criteria included male at birth and male gender identity, ≥15 years old, residing in the US, and reported oral or anal sex with a man at least once at any time in the past. Survey respondents were asked to provide an email address for future study invitations. Participants of the 2017 and 2018 data collection cycles who were at least 18 years of age and self-reported a positive HIV status were emailed an invitation to participate in the DBS VL study. After consenting to participate in the DBS collection and completing an online survey, participants were mailed a DBS collection kit containing written instructions, one Whatman 903 protein saver DBS card, two contact-activated lancets, two sterile gauze, two alcohol pads, two bandages, five desiccants, a humidity indicator card, one plastic zipper bag, and a pre-paid return envelope. In the online survey, participants answered questions about whether they missed any ARV doses in the past 4 and 30 days and how often they took ARV as directed in the past 30 days. Participants were considered ARV adherent if they didn’t report missing any doses in the past 4 days and reported “always” or “almost always” taking ARV as directed in the past 30 days. The electronic informed consent in the AMIS protocol included only VL testing.

Participants from both studies were instructed to write the date they collected their blood on the card and to follow the mailing instructions provided with the DBS collection kits.

### Testing on DBS

DBS from eSTAMP were first tested with the FDA-approved Avioq HIV-1 Microelisa System to detect antibody to HIV (Avioq) (Avioq, Inc., Rockville, MD) and, if repeatedly reactive, followed with GS HIV-1 Western Blot (WB) (Bio-Rad laboratories, Redmond, WA) for confirmation. Each test requires one 6-mm punch of DBS and were performed following package insert instructions [9, 10]. For DBS cards with sufficient spots, a modified GS HIV Combo Ag/Ab EIA (Ag/Ab) (Bio-Rad laboratories) DBS protocol was performed to detect HIV p24 antigen and antibody, followed by a modified Geenius HIV-1/2 Supplemental Assay (Geenius) (Bio-Rad laboratories) DBS protocol if repeatedly reactive. These two modified protocols were validated and published previously [11]. Due to the limited blood spots per card, we modified the Abbott RealTi*me* HIV-1 RNA DBS protocol [12] with a limit of detection (LOD) of 2.92 log_10_ (copies/mL) using a 12 mm perforated circle (~ 70 μL whole blood) to use four 6 mm punches of DBS (~ 50 μL whole blood) [13]. The validated 4-punch protocol showed a linearity agreement of R^2^ = 0.924 when comparing the DBS and 0.8 mL plasma protocols (unpublished data).

Concentrations of Tenofovir (TFV), Emtricitabine (FTC), Abacavir (ABC), Lamudivine (3TC), Efavirenz (EFV), and Raltegravir (RAL), in DBS were measured by high-performance liquid chromatography-tandem mass spectrometry (HPLC-MS) (Sciex, Foster City, CA). TFV/FTC as well as ABC/3TC capture the nucleoside analog backbones of commonly used antiretroviral regimens. The non-nucleoside reverse transcriptase inhibitor EFV was a component in some regimens at the time of sampling. The integrase inhibitor RAL is commonly added to nucleoside analog backbones in antiretroviral therapy. The procedure was described in our previous publication [13].

### Analyses

Using the DBS collected in eSTAMP, we compared the HIV serological results using FDA-approved protocols with laboratory-modified protocols to evaluate the feasibility of HIV testing using DBS. VL and ARV were measured to examine the correlation between VL levels and ARV presence in samples with enough quantity of spots and to fully characterize HIV-positive test results. The VL results from AMIS DBS were used to further assess the feasibility of using the DBS for monitoring viral suppression.

## Results

### Demographics of the participants who mailed back DBS cards

eSTAMP participants were recruited from across the US (38.7% South, 23.5% West, 19.3% Midwest, 18.5% Northeast) and most (69.5%) were between 18 and 34 years old. Participants were mostly non-Hispanic white (67.9%) followed by 19.6% Hispanic, 7.4% black, and 5.1% Asian.

AMIS study participants were from across the US as well and 74.4% were non-Hispanic white, 12.8% Hispanic, 7.7% black, and 5.1% others or multiple races. The majority (56.4%) of AMIS participants were over 50 years old.

### Description of the DBS collections

From May 2014 to December 2016, 2313 eSTAMP participants were mailed a DBS collection kit and 1456 (62.9%) returned self-collected cards to the CDC laboratory. Twelve cards were rejected for testing; the spots were too small for a single 6 mm punch (*n* = 6) or the participant’s unique identifier was not written on the card (n = 6). Of 1444 (98.0%) cards eligible for testing, the median time at ambient temperature from collection to arrival at the laboratory was 8 days; 1300 (90.0%) were received within 14 days, 93 (6.5%) from 14 to 21 days, and 51 (3.5%) more than 3 weeks from the collection date written on the card. Regarding the quantity of spots either partially or fully filled with blood, 1286 (89.1%) had five spots, 97 (6.7%) had four spots, 58 (4.0%) had three spots, and three cards (0.2%) had one or two spots filled. Based on the humidity indicator, 637 (44.1%) were exposed to high humidity (≥50%) and 38 (2.6%) did not have a humidity indicator upon arrival at the CDC laboratory. Of the remaining 769 (53.3%) that were within recommended storage condition for Avioq testing (< 50% humidity) [9], 525 (36.4%) and 244 (16.9%) were exposed to 40 and 30% humidity, respectively.

Out of 111 AMIS participants, 78 (70.3%) mailed self-collected DBS back to CDC laboratory for VL testing. Nearly half of them were missing the date of sample collection, therefore we calculated the median days from when the DBS cards were mailed to participants until their arrival at the CDC laboratory. The median number of days until arrival was 11.5 days (range 4 to 70 days) and 67.9% (53/78) of AMIS DBS cards were received within 14 days. All 78 had five spots either partially or fully filled with blood, which was sufficient for VL assay. Six cards (7.7%) were sent without humidity indicators. Ten (12.8%) and 62 (79.5%) were exposed to 40% and ≤ 30% humidity, respectively.

### Laboratory-based testing results

**Fig. 2:**
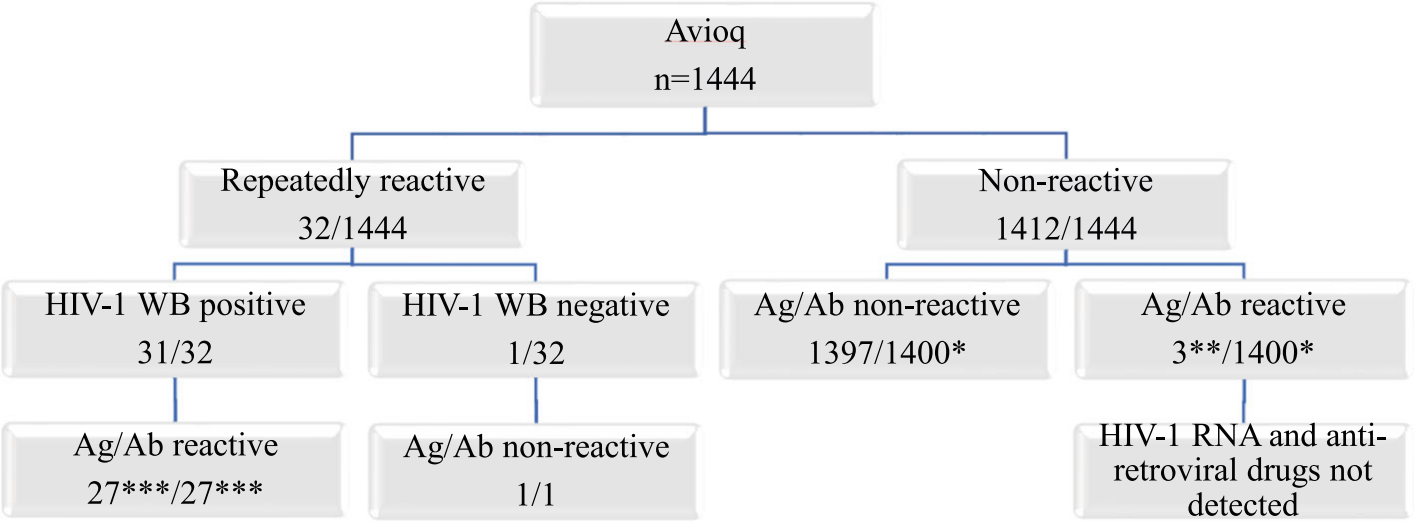
Laboratory-based testing results of eSTAMP specimens. *Abbreviations*: Avioq, Avioq HIV-1 Microelisa System; WB, western blot; Ag/Ab, Bio-Rad GS HIV Ag/Ab combo; Geenius, Bio-Rad HIV1/2 Geenius Supplemental. *Footnotes*: *12 samples were not tested with Ag/Ab due to insufficient DBS quantity. **Three samples had low signal cutoff ratio (1.2, 1.4,1.5) and were not repeated in duplicate with Ag/Ab protocol or tested with WB or Geenius because of insufficient DBS quantity. ***Four samples were not tested with Ag/Ab due to insufficient DBS quantity

Of 1444 cards tested with Avioq, 32 cards (2.2%) were repeatedly reactive (Fig. 2). Of those, 31 were confirmed as WB HIV-1 positive and one was WB HIV-1 negative. Of 31 Avioq-reactive/WB positive, 27 had sufficient spots for Bio-Rad Ag/Ab testing and all were reactive. The one Avioq-reactive/WB HIV-negative DBS was non-reactive by Bio-Rad Ag/Ab. Among 1400 Avioq-non-reactive DBS, Bio-Rad Ag/Ab was non-reactive in 1397 (99.8%), and three were initially reactive but were not repeated in duplicate due to insufficient quantity. The initial signal/cutoff ratio for the three samples were 1.2, 1.4, and 1.5, respectively, and all three had undetectable VL and ARV levels.

Nine of 27 reactive samples had sufficient quantity of spots for Geenius testing, VL, and ARV measurement by HPLC-MS (Table 1). All were Geenius HIV-1 positive, and two samples without detectable ARV had VL > 5 log_10_ (copies/mL). In contrast, of seven samples with presence of at least one ARV drug, five had undetectable VL and two had VL values of 3.29 and 3.58 log_10_ (copies/mL).

**Table 1:** Characterization of HIV-1 antibody positive dried blood spots from nine eSTAMP participants

Viral load log_10_ (copies/mL)	Humidity (%)	Days from collection to CDC	RAL	ABC	3TC	EFV	TFV	FTC
5.17	40	21	–	–	–	–	–	–
5.40	40	15	–	–	–	–	–	–
3.29	40	13	–	–	–	–	+	+
3.58	50	20	–	–	–	–	–	+
Target not detected	50	13	–	–	–	–	–	+
Target not detected	50	48	–	–	–	–	+	+
Target not detected	50	13	–	–	–	–	+	+
Target not detected	40	15	–	–	–	–	+/−	+
Target not detected	no indicator	11	–	–	–	+	+	+

*Abbreviations*: *RAL* Raltegravir, *ABC* Abacavir, *3TC* Lamudivine, *EFV* Efavirenz, *TFV* Tenofovir, *FTC* Emtricitabine

To further assess the use of the DBS for VL monitoring, VL results from the AMIS study were compared to self-reported ARV adherence. Of 78 DBS, 4 were excluded due to contradictory responses to questions about taking ARV medications, 63 had undetectable VL, seven had detectable but unquantifiable VL (< 2.92 log_10_ (copies/mL)), and four had quantifiable VL ranging from 3.04 to 5.30 log_10_ (copies/mL). Of the four with quantifiable VL, three were not ARV adherent and one was not on ARV treatment. Among the seven with detectable but unquantifiable VL, five participants were considered adherent; one missed doses on 2 of 4 days, and one didn’t record the ARV information in the survey. Table 2 lists the 11 detectable VL results and their self-reported adherence measures. Among 63 participants with undetectable VL, three missed doses on 2 of 4 days, six missed doses on 1 of 4 days, and one was not taking ARV.

**Table 2:** Characterization of dried blood spots from 11 AMIS participants with detectable HIV-1 viral load

Viral load log_10_ Copies/ml	Humidity (%)	Days between kits mailed and arrived at CDC	Days missed all doses, past 4 days	Days missed 1 dose or more, past 30 days	Taking ARV as directed, past 30 days (self-reported)
3.04	< 30	9	4	25	rarely
4.48	< 30	10	Not on ARV		
5.02	< 30	10	4	14	never
5.30	< 30	8	4	30	never
< 2.92	40	72	0	2	almost always
< 2.92	30	7	Not available^a^		
< 2.92	30	10	0	0	always
< 2.92	30	11	0	0	always
< 2.92	< 30	8	2	7	usually
< 2.92	< 30	9	0	0	always
< 2.92	no indicator	9	0	0	always

*Abbreviation*: *ARV* Antiretroviral

^a^The participant didn’t provide the ARV information in the online survey

## Discussion

We describe a large-scale laboratory assessment of DBS cards collected by MSM recruited into online HIV research studies in the US. Most MSM followed the DBS collection and packaging/mailing instructions to provide their own specimens for HIV laboratory-based testing. Our analyses support the self-collection and mailing of DBS as a possible strategy for collecting blood specimens from MSM for HIV testing and treatment monitoring [14, 15].

Almost all eSTAMP DBS had sufficient spots for obtaining a single 6 mm punch, which was adequate for Avioq testing. DBS from AMIS all had adequate spots for the VL assay. This is similar to a previous study [16] where DBS collected by non-medically trained interviewers showed 99% of the cards yielded enough sample for at least one analysis. Although our study recorded humidity exposure data, the effect of high humidity on testing results was not evaluated. Exposure to humidity might be reduced by increasing the number of desiccant packs (minimum five), emphasizing proper drying of samples, and complete sealing of the bag prior to packaging and shipping. An alternative DBS collection device with moisture-tight design, HemaSpot HF blood collection device (Spot On Sciences, San Francisco, CA), has been previously demonstrated [17]. The use of such a device could reduce humidity exposure and should be explored further in future large studies.

Overall, the Bio-Rad Ag/Ab performed similarly to the Avioq antibody test at detecting HIV positive samples. One Avioq-reactive/WB HIV-1 negative DBS was non-reactive with Bio-Rad Ag/Ab which may indicate Avioq false-reactivity. Three Avioq-non-reactive, but Bio-Rad Ag/Ab reactivity DBS had low signal/cutoff ratio. Due to insufficient quantity, the three DBS were not retested in duplicate as required by protocol to validate the initial results. Considering the low reactivity, undetectable VL and ARV levels, the initial Ag/Ab reactivity may be false-reactive.

It has been well established that DBS collected by trained professionals are suitable for VL assay [12, 18–20]. However, self-collected DBS for VL quantification has been explored only recently. A large study with MSM living with HIV demonstrated that 60.8% of enrolled MSM provided DBS with adequate spots for VL testing and 52.5% of those had detectable VL [14]. To our knowledge, our study is the first assessing both VL and ARV measurements using self-collected DBS from MSM. Despite the small number of eSTAMP DBS for VL and ARV testing, our results showed that DBS with undetectable ARV had higher VL than DBS with at least one detectable ARV. The association of high VL and self-reported poor ARV adherence was also observed in AMIS participants. However, none of the studies, including ours, performed a comparison of VL testing on DBS with matched plasma. Future studies are required to establish the correlation of VL values between self-collected DBS and plasma specimens.

A few limitations of our study should be recognized: (1) the small sample size of HIV-1 positive DBS, (2) possible compromised RNA integrity of the DBS exposed to high humidity, (3) higher LOD of the DBS VL protocol compared to plasma. Due to the limited specimen quantity and number of tests performed, we used a modified VL DBS protocol. One third of the 3 log_10_ (copies/ml) DBS panel were undetectable by our 4-punch protocol, which may indicate that the LOD of our protocol was higher than 2.92 log_10_ copies/ml published by Abbott Molecular [12]. Thus, samples with low VL may have been missed. Although our study did not observe invalid results in the DBS exposed to high humidity, studies with large sample size of DBS from people with HIV are needed to evaluate the impact of high humidity exposure on HIV testing using self-collected DBS, (4) the six ARV drugs measured in our study were among the most commonly used at the time of data collection (2014–2016), however, there was a possibility that participants may receive other ARVs, and (5) participants were predominately non-Hispanic white men, therefore the self-collected DBS approach needs to be further evaluated in future studies with more diversified participants.

## Conclusions

MSM from two online studies were able to successfully self-collect DBS for laboratory testing. The modified Bio-Rad Ag/Ab protocol performed similarly to the FDA-approved Avioq protocol for antibody detection and concordance between the testing algorithms was high. Our study demonstrated that DBS self-collected by MSM is feasible for HIV testing, ARV measurement, and VL monitoring.

## Data Availability

The datasets used and/or analyzed in the study are available from the corresponding author on reasonable request.

## Abbreviations

3TC: Lamudivine
ABC: Abacavir
Ag/Ab: GS HIV Combo Ag/Ab EIA
AMIS: American Men’s Internet Survey
ARV: Antiretroviral
Avioq: Avioq HIV-1 Microelisa System
CDC: Centers for Disease Control and Prevention
DBS: Dried blood spots
EFV: Efavirenz
eSTAMP: Evaluation of HIV Self-Testing Among MSM Project
FDA: Food and Drug Administration
FTC: Emtricitabine
Geenuis: Geenius HIV-1/2 Supplemental Assay
HPLC-MS: High-performance liquid chromatography-tandem mass spectrometry
LOD: Limit of detection
MSM: Men who have sex with men
RAL: Raltegravir (RAL)
RCT: Randomized controlled trial
TFV: Tenofovir
US: United States
VL: Viral load
WB: GS HIV-1 Western Blot
